# Does getting online help low-income adults eat better? Digital access and BMI-based nutritional imbalance in China

**DOI:** 10.3389/fnut.2026.1927357

**Published:** 2026-09-28

**Authors:** Zuocheng Li, Xiaoning Ma, Xue Lei

**Affiliations:** 1 School of Law, Huazhong University of Science and Technology, Wuhan, China; 2 School of Discipline Inspection and Supervision, Inner Mongolia University, Hohhot, China; 3 School of Management, Shanghai University, Shanghai, China

**Keywords:** body mass index, digital access, food security, low-income populations, nutritional epidemiology, nutritional imbalance

## Abstract

**Introduction:**

The digital economy has reshaped how people work, shop, and manage health, yet uneven nutrition remains a pressing livelihood concern for low-income groups in China now that absolute poverty has ended. Most of what is known about improving nutrition in these households has to do with money, whether cash transfers or food subsidies, and much less is known about what changes when a household simply gets online. This study asks whether being connected goes together with a lower risk of nutritional imbalance among low-income adults, and what may lie behind that link.

**Methods:**

Nutritional imbalance here covers both sides of the problem, being too thin and being too heavy, since low-income households in China face the two at once. It is built from body mass index and coded as six ordered categories, with a higher value standing for a more serious departure from a healthy weight. We draw on five waves of the China Family Panel Studies covering 2014 to 2022 and focus on adults aged 18 to 69 whose household per capita income falls in the bottom fifth within their province, which leaves 12,076 observations. The estimates compare the same person across waves, setting aside what is fixed about an individual and what moves for everyone at once. Three possible routes are then examined in turn, namely the weight of food in the household budget, labor income, and exercise frequency.

**Results:**

Low-income adults who are online carry a lower risk of nutritional imbalance, and the finding holds when the income line is drawn differently, when connectivity is counted differently, and when the model is changed. Being online goes together most clearly with more regular exercise, and it also goes together with a slightly larger share of the household budget spent on food and with higher earnings among those who work. The link is strongest for rural residents, for adults aged 50 and over, and for those who left school at or before the end of lower secondary education, while among urban, younger, and better educated adults no clear link shows up.

**Discussion:**

Connectivity seems to supplement offline provision rather than replace it, since little shows up where offline services are already dense. Expanding rural digital infrastructure and pairing online with offline nutrition support may therefore help narrow the nutrition gap that runs alongside the digital divide. The findings are reported throughout as associations.

## 1 Introduction

The United Nations Sustainable Development Goals call for ending all forms of hunger and malnutrition and achieving food security and improved nutrition for all by 2030 (1). Progress toward that goal has been uneven, and nutritional imbalance remains a major risk for low-income groups in particular. Compared with high-income groups, low-income populations lack the resources to absorb economic fluctuations, health crises, or rising prices, so their diets deteriorate more readily and their intake becomes inadequate or poorly balanced (2). The consequences reach well beyond the dinner table. As a visible manifestation of multidimensional poverty, nutritional imbalance undermines physical and mental health and cognitive development, impedes the accumulation of human capital, and can pass poverty from one generation to the next, holding back inclusive growth and social equity (3, 4).

China illustrates the problem clearly. Although absolute poverty has been eradicated, relative poverty persists, particularly in rural areas and in underdeveloped regions in the central and western parts of the country. Dietary quality among low-income groups has improved more slowly than income, and hidden hunger and structural malnutrition remain prominent (5). Easing nutritional imbalance among low-income populations and strengthening their dietary resilience has therefore become a major practical challenge in the post-poverty-alleviation era.

Work on how to ease nutritional imbalance has tended to look at macro-level policy. Minimum living security schemes, nutritional subsidy programmes, and rural health insurance have all been linked to better nutritional status among low-income groups (6–8). Yet these instruments share a common shape. Social relief and transfer payments compensate after the fact, they do little about the deeper constraints that low-income groups face in reaching information, markets, and equal opportunities, and they run into limited targeting accuracy and welfare dependency along the way (9). That gap is what makes digital technology worth a closer look. The internet, smartphones, and inclusive digital finance are reshaping social production and lifestyles on a wide scale (10), and in doing so they offer a way around the temporal and spatial barriers of traditional resource allocation that may empower vulnerable groups (11). Whether digital access can serve as a new lever for easing nutritional imbalance among low-income populations therefore holds both theoretical value and practical urgency.

Several lines of reasoning suggest it might. The first concerns information. Digital access lowers the cost of searching and helps break down information silos (12), which matters for a group constrained by education and social networks, short on scientific knowledge of nutrition and health, and easily swayed by low-priced, low-quality food products (13). Once online, people can reach dietary guidelines, knowledge of healthy eating, and information on food prices, all of which support better consumption decisions and greater dietary diversity (14). The second concerns money. Digital access broadens income-generating channels and reduces transaction costs (15), since digital technology opens the way to non-agricultural employment and entrepreneurial opportunities that raise household income (16, 17), while e-commerce and digital payments lower the cost of buying food and put a wider variety of ingredients within reach at lower prices (18, 19). The third concerns social support. Digital access widens the boundaries of social networks, so that low-income groups can more readily seek help from relatives and friends or reach mutual aid when a shock hits, which smooths consumption and keeps nutritional status from deteriorating sharply (20).

Despite a growing body of work on how digital technologies relate to residents' health and welfare, research on digital access and nutritional imbalance remains limited, and four gaps stand out. First, in terms of concept, most studies look at static outcomes such as subjective wellbeing, healthcare expenditure, or single physiological indicators (21, 22), or stop at dietary diversity and food expenditure, paying less attention to nutritional risk as a state that spans both undernutrition and overnutrition and that a household's own coping capacity can buffer. Second, in terms of sample, most studies treat residents as a homogeneous group, which passes over the dual economic and information constraints that low-income groups face and leaves the safety-net role of digital access for this population unclear. Third, in terms of data, cross-sectional designs dominate, which makes it hard to account for differences between individuals and for change over time. Fourth, in terms of routes, household or individual digital access reflects the actual capacity of micro-level actors to reach and use digital technologies more directly than macro-level indicators of digital economic development do (23), yet whether the link runs through information, income, or health has rarely been examined. Taken together, existing research has not brought micro-level digital access, low-income populations, and nutritional imbalance into a single framework, nor clarified the routes along which the link operates.

China is a suitable setting in which to fill those gaps. In recent years the country's digital infrastructure has developed rapidly and the digital divide has been narrowing steadily. As of June 2023 the number of internet users stood at 1.079 billion, the national penetration rate reached 76.4%, and the rural penetration rate exceeded 60% (24, 25). At the same time, China has built a very large social security system that still struggles to improve the nutritional intake of low-income populations and to keep them from slipping back into poverty. Asking whether and how digital access eases nutritional imbalance in this setting therefore offers both a reflection on poverty governance in the digital age and a Chinese perspective on rural revitalization and the Sustainable Development Goals. Drawing on five waves of the China Family Panel Studies, this study assesses the link between digital access and nutritional imbalance among low-income populations, traces the routes along which it runs, and examines how the pattern varies across residence, age, and schooling.

Against the existing literature, and answering the four gaps in turn, the contributions of this paper lie in four aspects. First, it brings digital access and nutritional imbalance into a single analytical framework, moving beyond studies that looked only at static nutritional outcomes or at income poverty, and it points to new ways in which digital technologies can empower vulnerable groups. Second, it works with a sample of low-income groups alone, which removes the confounding presence of better-off households and gives a sharper picture of the safety-net role of digital access. Third, it builds an integrated framework covering information, economic, and health routes, and reports evidence on each rather than asserting any of them. Fourth, it follows the same individuals across five waves and checks the findings in several ways, which strengthens the reliability and policy relevance of what it reports. The results offer micro-level evidence on the role of digital technology in inclusive development and a basis for designing digital support and nutrition programmes aimed at low-income populations.

## 2 Theory and hypotheses

### 2.1 Dual constraints on nutrition

Nutritional imbalance here refers to the risk that a household's nutritional status deteriorates when a shock arrives, together with a limited capacity to cope with that shock. Unlike static indicators of malnutrition, it turns on how likely a future nutritional crisis is and how severe it would be (26). Seen through sustainable livelihood theory and the vulnerability framework, that risk arises from the interplay between exposure, sensitivity, and adaptive capacity. For low-income populations two constraints do most of the work. The first is economic. Given limited income, low-income households are often forced to cut food spending when prices move, work dries up, or someone falls ill, which lowers dietary quality and puts nutrient-dense foods out of reach (27). The second is informational. Limited schooling and thin social networks leave these households at a disadvantage in reaching nutritional knowledge and health information, which makes sound dietary decisions harder and leaves them exposed to hidden hunger (28).

The two constraints compound each other. A well-documented regularity in food demand holds that as income rises the share of starchy staple foods in the diet declines while the share of high-nutrient foods such as animal proteins increases (29). The dietary structure of low-income groups is therefore sensitive to changes in income, and even minor fluctuations can produce a sharp decline in dietary diversity. Higher income supplies the material foundation for better nutritional status, while information and social support serve as the softer supports for resilience (30). This is precisely where digital technology may enter, since by reshaping the flow of information, financial resources, and social connections it can ease both constraints at once and change how households cope with nutritional risk (31).

### 2.2 Three routes of digital access

Drawing on information economics, social capital theory, and consumer economics, this paper sets out three routes along which digital access may ease nutritional imbalance, namely information empowerment, economic empowerment, and health protection. Each takes one of the constraints described above as its starting point.

#### 2.2.1 Information empowerment

In traditional settings a knowledge barrier surrounds nutritional and health information. Constrained by education and living conditions, low-income groups often lack scientific knowledge of balanced diets, micronutrient supplementation, and food safety. Their dietary behavior runs on habit or on the appeal of low prices, which produces a poor-quality diet marked by high carbohydrate intake and low protein and vitamin intake (32, 33). Information-search theory holds that the cost of obtaining information shapes the quality of the decisions people make, and digital access has cut that cost sharply, opening channels through which low-income groups can reach a large body of health information.

Two things follow. The spread of smartphones and the internet has broken down spatial and hierarchical barriers, so that low-income groups can reach professional medical advice, nutritional science, and dietary guidelines through short videos, health applications, and social media, which helps correct misconceptions and raises nutritional literacy (34). Watching instructional videos, for instance, lets people learn which inexpensive ingredients combine into a nutritionally adequate meal, rather than reading price alone as a signal of what to buy. Digital access also breaks down information silos, so that news of food price movements and nutritional subsidy policies arrives in time to be acted on, which supports better food purchases within a fixed budget. Broader access to information has already been linked to stronger health literacy and more preventive health behavior among rural residents (35).

#### 2.2.2 Economic empowerment

Information helps only so far when the budget will not stretch, which brings the second route into view. Income is the most direct factor shaping dietary quality, and limited purchasing power is a central reason why low-income groups cannot afford a varied, high-quality diet. Digital access opens new avenues for raising income by reshaping employment patterns and the distribution of earnings (36). Through digital platforms, low-income individuals can work around geographical constraints and take part in non-agricultural employment or the gig economy, including ride-hailing, food delivery, and e-commerce customer service, which raises wage income (37). Rural e-commerce and social media marketing let low-income farmers connect their produce more directly to larger markets, which cuts the profit taken by intermediaries and improves their ability to secure better prices and higher sales (38). Digital inclusive finance has eased credit constraints alongside this, since digital payments and microcredit lower financing barriers and let households invest in health-related assets or absorb unexpected expenses without cutting food spending (39).

What this buys a household is both a better diet and a steadier one. Higher income shifts food consumption from simply satisfying hunger toward better-quality food, with greater intake of high-protein and high-vitamin items. It also provides a buffer against price and income shocks, so that a household facing external risk is less likely to cut food spending sharply or let dietary quality slip.

#### 2.2.3 Health protection

A third route runs through health itself, because nutritional status and health status reinforce each other. A decline in health impairs the body's ability to absorb and use nutrients, and it raises nutritional risk indirectly as healthcare spending crowds out the food budget. For low-income groups, health crises often mark a turning point, and the risk is more pronounced given their limited capacity for health management and their weaker access to care. Digital access builds a digital health shield across three dimensions, namely access to health information, disease prevention, and access to healthcare (40).

On the prevention side, mobile internet lets low-income individuals keep track of their health and pick up early warnings through health applications, wearable devices, and online consultation platforms, so that minor ailments are addressed before they develop into serious conditions that deplete nutritional reserves. Digital health records and chronic disease management tools also build awareness of regular medication and dietary adjustment, which reduces the metabolic disturbance that follows poor disease control. On the access side, internet hospitals and telemedicine reduce geographical barriers to quality care, so that residents in remote areas can obtain professional advice at lower cost and delayed diagnosis becomes less common (41). When health crises do occur, timely intervention shortens the illness and lowers the risk of complications, which limits the squeeze that medical spending places on the food budget.

Based on the analysis above, the following hypotheses are proposed.

**H1:** Digital access is associated with a lower likelihood of nutritional imbalance defined by body mass index (BMI) among low-income populations.

**H2:** The association between digital access and nutritional imbalance among low-income populations runs partly through information empowerment.

**H3:** The association between digital access and nutritional imbalance among low-income populations runs partly through economic empowerment.

**H4:** The association between digital access and nutritional imbalance among low-income populations runs partly through health protection.

Figure 1 sets out the hypothesized structure, with the direct route and the three indirect routes shown side by side.

**Figure 1:**
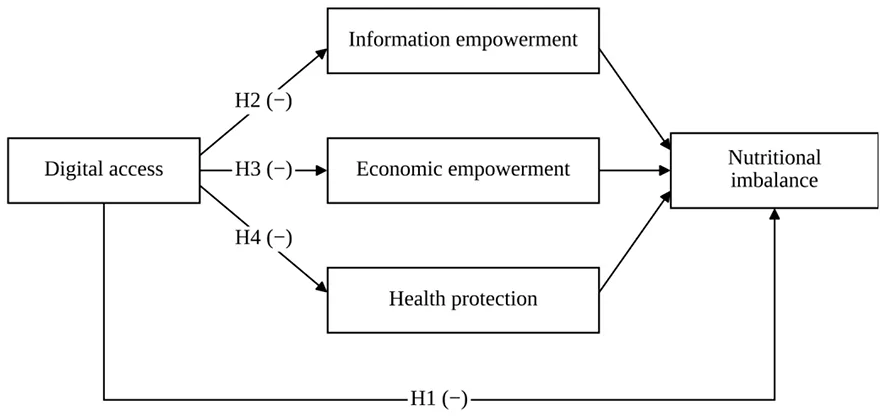
Research framework. Digital access relates to nutritional imbalance directly (H1) and through three routes, namely information empowerment (H2), economic empowerment (H3), and health protection (H4). Arrows run from the antecedent to the consequent, and the sign in parentheses is the hypothesized direction of each route on the nutritional imbalance index.

## 3 Data and variables

### 3.1 Data sources

The data come from the China Family Panel Studies, conducted by the Institute of Social Science Survey at Peking University. Five waves are used, covering 2014, 2016, 2018, 2020, and 2022. The survey uses a multi-stage, multi-strata probability sampling design covering 25 provinces, municipalities, and autonomous regions, and it collects data at the individual, household, and community levels. The sample is nationally representative and follows the same people over time, which is what allows income, consumption, health, and digital use to be traced together. Three selection rules were applied. First, the sample was limited to adults aged 18 to 69, which leaves out minors whose diets are chosen at the household level and the oldest adults whose weight shifts for reasons of aging rather than diet. Second, low income was defined as a household whose per capita income in the survey year fell within the lowest 20th percentile in its province. Drawing the line province by province and year by year, rather than once nationally, respects uneven regional development and the wide disparities in prices and incomes across provinces, and it avoids understating the size of low-income groups in the central and western regions and in rural areas. Third, during cleaning, observations with missing values or logical anomalies on the outcome, on digital access, or on per capita household income were deleted, and the top and bottom 1% of household consumption, healthcare expenditure, and assets were truncated. Provincial food consumer price index (CPI) and regional internet penetration were then matched to individual records by province and year, with the macro-level series drawn from successive editions of the China Statistical Yearbook and from provincial statistical yearbooks. These rules leave 12,076 observations. The number of people present varies from wave to wave, since low-income status is judged afresh each year and a household enters or leaves as its relative position moves, and since some respondents drop out or skip questions along the way.

### 3.2 Variables and measurement

#### 3.2.1 Dependent variable

Nutritional imbalance (NUTRIIMB) is built from body mass index. The literature measures nutritional status in two ways. One relies on 24-h dietary recalls and detailed food consumption records to compute energy and micronutrient gaps. The other groups individuals into underweight, normal weight, overweight, and obese categories on the basis of body mass index. Dietary recall is the more direct instrument, but in large-scale surveys it carries substantial measurement error and captures what someone happened to eat that day rather than how they eat over the long run. Body mass index accumulates the longer-run consequences of diet and health in a single comparable number. The survey questionnaire does not collect detailed dietary intake, so respondents are classified into six ordered categories, coded as normal weight = 0, overweight = 1, obese = 2, mildly underweight = 3, moderately underweight = 4, and severely underweight = 5, where a higher value stands for a more serious departure from a healthy weight. The ordering places the three underweight grades above overweight and obesity because in a low-income population wasting carries the more immediate health penalty and the more direct link to inadequate energy and protein intake, while overweight in the same population reflects a diet skewed toward cheap energy-dense staples. The indicator therefore covers both undernutrition and overnutrition, going beyond single underweight measures that capture only the risk of hunger.

#### 3.2.2 Independent variable

Digital access (INTERNET) is coded 1 if the respondent reports using the internet on any device in the survey year, and 0 otherwise. Mobile internet access (MOBNET) is coded 1 if the respondent reports going online with a mobile device. The second variable is a subset of the first, since 19.8% of the sample goes online by mobile against 24.7% who go online at all, so roughly four in five of those online use a phone.

#### 3.2.3 Control variables

At the individual level the controls are years of schooling (EDUYR), marital status (MARRIED), employment status (EMPLOYED), self-rated health (SRH), chronic disease (CHRONIC), insurance coverage (INSURED), and rural household registration (RURALHK). At the household level they are household size (FAMSIZE), the logarithm of per capita household income (LNINCPC), the logarithm of per capita household food expenditure (LNFOODPC), the logarithm of household net assets (LNASSET), the share of children (CHILDRATIO), the share of elderly members (ELDRATIO), the logarithm of household healthcare expenditure (LNMEDEXP), and agricultural operation (AGRIOP). Age (AGE) and sex (MALE) appear in the descriptive statistics but not among the regressors, since age equals the year minus the year of birth and is absorbed once individual and year fixed effects are in place, while sex does not vary within a person.

#### 3.2.4 Route variables

Three variables stand for the three routes. The weight of food in the budget (FOODSHARE) is household food expenditure divided by total household consumption expenditure. Labor income (LNLABINC) is the logarithm of individual total labor income, recorded only for respondents who report positive earnings. Exercise frequency (EXERCISE) is the number of times per week the respondent reports taking physical exercise. The information route of H2 has no variable of its own in the survey, so it is read here through the two variables that respond to better information, namely how the budget is allocated and how often people exercise.

Descriptive statistics are reported in Table 1.

**Table 1:** Descriptive statistics of main variables.

Variable	*N*	Mean	SD	Min	Median	Max
NUTRIIMB	12,076	0.527	0.965	0.000	0.000	5.000
INTERNET	12,076	0.247	0.431	0.000	0.000	1.000
MOBNET	12,076	0.198	0.399	0.000	0.000	1.000
AGE	12,076	48.811	13.652	18.000	50.000	69.000
MALE	12,076	0.484	0.500	0.000	0.000	1.000
EDUYR	12,076	5.560	4.414	0.000	6.000	19.000
MARRIED	12,076	0.885	0.320	0.000	1.000	1.000
EMPLOYED	12,076	0.840	0.367	0.000	1.000	1.000
SRH	12,076	2.846	1.321	1.000	3.000	5.000
CHRONIC	12,076	0.179	0.384	0.000	0.000	1.000
INSURED	12,076	0.969	0.172	0.000	1.000	1.000
RURALHK	12,076	0.924	0.265	0.000	1.000	1.000
FAMSIZE	12,076	4.748	2.310	1.000	5.000	21.000
LNINCPC	12,076	7.950	1.037	0.000	8.142	10.309
LNFOODPC	12,076	7.462	1.119	0.000	7.601	10.309
LNASSET	12,076	11.705	1.227	0.000	11.792	17.731
CHILDRATIO	12,076	0.199	0.185	0.000	0.200	1.000
ELDRATIO	12,076	0.262	0.312	0.000	0.200	1.000
LNMEDEXP	12,076	6.889	2.630	0.000	7.601	13.122
AGRIOP	12,076	0.900	0.299	0.000	1.000	1.000

Every observation satisfies the bottom-fifth provincial income rule by construction, so no indicator for low-income status is reported.

Three features of Table 1 are worth drawing out. The outcome has a mean of 0.527 against a median of 0, so most people sit at normal weight while a sizeable minority is spread across the five risk categories. Digital access is far from universal, which leaves plenty of movement in and out of connectivity for the estimates to work with. And schooling averages 5.560 years while 92.4% of the sample holds rural household registration, which confirms that the sample is drawn from the part of the population where both constraints described earlier bind hardest.

### 3.3 Empirical strategy

#### 3.3.1 Baseline model

People differ from one another in ways that are hard to observe, and everyone is exposed to the same national trends, so a simple comparison between those online and those offline would confound both. The baseline therefore compares the same person with himself or herself across waves, net of what all respondents experienced in a given year:


$$\begin{array}{c} NUTRIIMB_{it}=\beta_{0}+\beta_{1}INTERNET_{it}+\gamma \prime X_{it}+\mu_{i}+\lambda_{t}+\varepsilon_{it} \end{array}$$ (1)

Here *NUTRIIMB*_*it*_ is the nutritional imbalance index of individual *i* in year *t*, with a higher value standing for greater risk, and *INTERNET*_*it*_ is the core explanatory variable. The coefficient β_1_ is the parameter of interest, and a negative value indicates that digital access goes together with a lower index. *X*_*it*_ collects the individual and household controls listed above, μ_*i*_ absorbs what is fixed about a person, such as genetic endowment and long-standing dietary habits, λ_*t*_ absorbs what moves for everyone at once, such as macroeconomic fluctuation and food price change, and ε_*it*_ is the error term. Standard errors are clustered at the individual level.

One caveat attaches to the control set. Two of the controls, LNINCPC and LNFOODPC, sit close to the economic route, so holding them fixed could in principle absorb part of what the core variable is meant to show. The baseline is therefore estimated both without and with the full control set, which lets the consequence of that choice be read directly.

#### 3.3.2 Route model

Establishing the overall link still leaves open where it comes from. To trace the three routes, each route variable is regressed on digital access under the same structure as the baseline:


$$M_{it}=\alpha_{0}+\alpha_{1}INTERNET_{it}+\delta^{\prime }X_{it}+$$ (2)

where *M*_*it*_ takes FOODSHARE, LNLABINC, or EXERCISE in turn. A coefficient α_1_ that differs from zero indicates that digital access and that route variable move together within individuals over time. The three results are read alongside the baseline rather than combined into a single decomposition, since the three route variables sit on different scales and are observed on partly different groups of people. When FOODSHARE is the dependent variable, LNFOODPC is dropped from the control set, because household food expenditure is what the dependent variable is built from.

## 4 Results

### 4.1 Connectivity and the imbalance index

Table 2 reports the baseline. Column (1) includes only digital access and the two sets of fixed effects, and column (2) adds the full control set. In column (1) the coefficient on digital access is −0.080 and differs from zero at the 5% level. In column (2) it is −0.085 and differs from zero at the 1% level. The estimate moves by 0.005 once 15 individual and household controls enter, which settles the caveat raised about the control set, since holding household income and food spending fixed turns out to make almost no difference to what the core variable shows.

**Table 2:** Baseline results.

Variable	(1)	(2)
	NUTRIIMB	NUTRIIMB
INTERNET	−0.080^**^	−0.085^***^
	(−2.544)	(−2.734)
EDUYR		−0.012
		(−0.987)
MARRIED		0.163^*^
		(1.722)
EMPLOYED		−0.058
		(−1.188)
SRH		−0.014
		(−1.064)
CHRONIC		0.037
		(1.070)
INSURED		0.079
		(1.066)
RURALHK		−0.165^**^
		(−2.326)
FAMSIZE		0.003
		(0.271)
LNINCPC		0.008
		(0.403)
LNFOODPC		0.007
		(0.543)
LNASSET		−0.000
		(−0.007)
CHILDRATIO		−0.094
		(−0.761)
ELDRATIO		−0.018
		(−0.179)
LNMEDEXP		−0.015^***^
		(−2.687)
AGRIOP		−0.070
		(−0.691)
Observations	12,076	12,076
R-squared	0.846	0.846
Individual FE	YES	YES
Year FE	YES	YES

Cluster-robust *t*-statistics at the individual level in parentheses. ^***^p < 0.01, ^**^p < 0.05, ^*^p < 0.1.

In practical terms, low-income adults who are online score about 0.085 units lower on the six-point index than the same people score in waves when they are not online. Against a sample mean of 0.527 that is a shift of roughly one sixth of the average level, and against a standard deviation of 0.965 it is about one twelfth. The magnitude is modest, which is what one would expect from simply being connected rather than from a nutrition programme, and it supports H1. Among the controls in Table 2, rural household registration carries −0.165 and household healthcare expenditure carries −0.015, both clearly different from zero, while marital status carries 0.163 at the 10% level.

### 4.2 Robustness tests

A finding of this kind can rest on three fragile choices, namely where the income line is drawn, what counts as being online, and how the six categories of the outcome are treated. Each is tested in turn.

#### 4.2.1 Moving the income line

The baseline draws the line at the 20th percentile of provincial income. Table 3 redraws it at the 30th and the 40th percentiles and estimates the baseline again on the enlarged samples. At the 30th percentile the coefficient is −0.085 and at the 40th it is −0.075, both clearly different from zero at the 1% level. The point estimate drifts toward zero as the line moves up, which is what the two constraints described earlier would lead one to expect, since households nearer the middle of the distribution face them less tightly and have less to gain from getting connected. The sample roughly doubles between the two columns of Table 3, so the result is not an artifact of where the line happens to sit.

**Table 3:** Moving the low-income threshold.

Variable	(1)	(2)
	NUTRIIMB	NUTRIIMB
	30th percentile	40th percentile
INTERNET	−0.085^***^	−0.075^***^
	(−3.736)	(−4.220)
EDUYR	0.009	0.008
	(0.938)	(1.073)
MARRIED	0.093	0.068
	(1.186)	(1.059)
EMPLOYED	−0.109^***^	−0.090^***^
	(−2.897)	(−3.049)
SRH	−0.018^*^	−0.019^***^
	(−1.925)	(−2.577)
CHRONIC	0.033	0.033
	(1.266)	(1.585)
INSURED	0.108^**^	0.027
	(2.017)	(0.625)
RURALHK	−0.108^*^	−0.016
	(−1.825)	(−0.371)
FAMSIZE	−0.001	−0.009
	(−0.136)	(−1.235)
LNINCPC	0.007	0.011
	(0.581)	(1.020)
LNFOODPC	−0.005	−0.000
	(−0.443)	(−0.014)
LNASSET	0.006	0.006
	(0.506)	(0.633)
CHILDRATIO	−0.113	−0.062
	(−1.301)	(−0.918)
ELDRATIO	−0.055	0.002
	(−0.807)	(0.048)
LNMEDEXP	−0.007^*^	−0.005
	(−1.807)	(−1.495)
AGRIOP	0.032	0.048
	(0.425)	(0.902)
Observations	19,483	26,941
R-squared	0.805	0.792
Individual FE	YES	YES
Year FE	YES	YES

Cluster-robust *t*-statistics at the individual level in parentheses. ^***^p < 0.01, ^**^p < 0.05, ^*^p < 0.1.

#### 4.2.2 Narrowing to mobile access

The baseline counts anyone who goes online as connected, whatever the device. Since rural and low-income groups in China now reach the internet mainly through phones, Table 4 replaces the broad marker with MOBNET, leaving the specification, the controls, and the fixed effects untouched. The coefficient is −0.086 and differs from zero at the 1% level, against −0.085 in the baseline.

**Table 4:** Narrowing the access measure to mobile devices.

Variable	(1)
	NUTRIIMB
MOBNET	−0.086^***^
	(−2.776)
EDUYR	−0.012
	(−0.990)
MARRIED	0.163^*^
	(1.721)
EMPLOYED	−0.058
	(−1.188)
SRH	−0.014
	(−1.066)
CHRONIC	0.037
	(1.073)
INSURED	0.079
	(1.067)
RURALHK	−0.165^**^
	(−2.332)
FAMSIZE	0.003
	(0.269)
LNINCPC	0.008
	(0.404)
LNFOODPC	0.007
	(0.543)
LNASSET	−0.000
	(−0.007)
CHILDRATIO	−0.093
	(−0.755)
ELDRATIO	−0.018
	(−0.179)
LNMEDEXP	−0.015^***^
	(−2.687)
AGRIOP	−0.070
	(−0.689)
Observations	12,076
R-squared	0.846
Individual FE	YES
Year FE	YES

Cluster-robust *t*-statistics at the individual level in parentheses. ^***^p < 0.01, ^**^p < 0.05, ^*^p < 0.1.

How close the two estimates sit deserves a word of caution. Mobile users are a subset of those online, and roughly four in five of those online use a phone, so the two markers describe largely the same people and their agreement says nothing about whether the device matters. What it does show is that the result is carried by the mobile majority rather than by the smaller group who reach the internet some other way, so the broader coding is not doing any work of its own.

#### 4.2.3 Respecting the ordered scale

The baseline treats the six codes as evenly spaced, which the coding rule does not guarantee. Table 5 therefore re-estimates the model in a way that uses only the ranking of the categories and not the distance between them:

**Table 5:** Pooled ordered logit.

Variable	(1)
	NUTRIIMB
INTERNET	−0.316^***^
	(−3.695)
EDUYR	−0.038^***^
	(−3.732)
MARRIED	−0.094
	(−0.748)
EMPLOYED	−0.366^***^
	(−3.670)
SRH	−0.146^***^
	(−5.076)
CHRONIC	0.188^**^
	(2.185)
INSURED	0.143
	(0.747)
RURALHK	−0.389^***^
	(−2.960)
FAMSIZE	−0.042^**^
	(−2.089)
LNINCPC	0.069^*^
	(1.732)
LNFOODPC	−0.018
	(−0.600)
LNASSET	−0.033
	(−1.185)
CHILDRATIO	−0.097
	(−0.423)
ELDRATIO	−0.212
	(−1.291)
LNMEDEXP	−0.024^*^
	(−1.894)
AGRIOP	0.082
	(0.595)
Observations	12,076
Province FE	YES
Year FE	YES

Cluster-robust *z*-statistics at the individual level in parentheses. ^***^p < 0.01, ^**^p < 0.05, ^*^p < 0.1.


$$\mathrm{Pr}\left(NUTRIIMB_{it}\leq j\right)=\Lambda \left(K_{j}-\beta_{1}INTERNET_{it}-\gamma^{\prime }X_{it}-\phi_{p}-\lambda_{t}\right),j=0,\ldots ,4$$ (3)

where Λ(·) is the logistic distribution function, κ_*j*_ are the cut points, and ϕ_*p*_ denotes province fixed effects. Individual fixed effects give way to province fixed effects here for a practical reason: an ordered logit carrying several thousand individual intercepts drops everyone whose category never changes, which would leave a very different set of people from the one behind the linear results. The coefficient on digital access is −0.316 and differs from zero at the 1% level, so being online goes with a lower chance of sitting in a higher risk category once only the ranking is used. Several controls that carry no weight under individual fixed effects turn up clearly different from zero in Table 5, including schooling at −0.038 and self-rated health at −0.146, which follows from the fact that the near-permanent part of those variables is no longer absorbed.

### 4.3 Tracing the three routes

The result so far says that being online and eating well go together, but not why. Table 6 takes each of the three routes in turn.

**Table 6:** Route regressions.

Variable	(1)	(2)	(3)
	FOODSHARE	LNLABINC	EXERCISE
INTERNET	0.014^*^	1.181^*^	0.270^**^
	(1.893)	(1.782)	(2.021)
EDUYR	−0.002	−0.223	0.031
	(−0.571)	(−0.898)	(0.629)
MARRIED	−0.012	−0.231	0.043
	(−0.634)	(−0.169)	(0.111)
EMPLOYED	−0.013	0.705	0.247
	(−1.318)	(1.294)	(1.583)
SRH	0.002	−0.018	0.037
	(0.417)	(−0.083)	(0.863)
CHRONIC	−0.011^*^	−0.460	0.457^***^
	(−1.688)	(−0.889)	(3.518)
INSURED	0.013	−0.423	−0.239
	(0.842)	(−0.647)	(−0.812)
RURALHK	0.004	0.440	−0.145
	(0.209)	(0.759)	(−0.460)
FAMSIZE	0.007^**^	0.674^***^	−0.087^**^
	(2.514)	(3.055)	(−2.051)
LNINCPC	−0.021^***^	0.891^**^	−0.211^***^
	(−4.918)	(2.316)	(−3.003)
LNFOODPC		0.113	−0.037
		(0.906)	(−0.878)
LNASSET	−0.008^***^	−0.292^**^	0.036
	(−2.671)	(−2.033)	(0.773)
CHILDRATIO	0.033	3.375^***^	0.530
	(1.242)	(2.969)	(1.369)
ELDRATIO	0.041^**^	0.340	0.128
	(2.216)	(0.413)	(0.359)
LNMEDEXP	−0.018^***^	−0.069	0.063^***^
	(−13.442)	(−0.853)	(3.240)
AGRIOP	0.029	−0.317	−0.451
	(1.428)	(−0.585)	(−1.268)
Observations	11,519	1,128	11,901
R-squared	0.802	0.973	0.863
Individual FE	YES	YES	YES
Year FE	YES	YES	YES

Cluster-robust *t*-statistics at the individual level in parentheses. ^***^p < 0.01, ^**^p < 0.05, ^*^p < 0.1. LNFOODPC is retained as a control in columns (2) and (3) but omitted from column (1), where household food expenditure is what the dependent variable is built from. Column (2) covers only observations reporting positive labor income.

#### 4.3.1 Weight of food in the budget

Column (1) of Table 6 puts the coefficient at 0.014 with a t-statistic of 1.893, which reaches the 10% level. Food takes about 1.4 percentage points more of the household basket in waves when the respondent is online. The figure is small because two forces pull against each other. Going online widens the range of food a household can find and compare, which pushes the food weight up, while it also lifts income and opens up non-food spending, which pushes the weight down. What remains is a mild tilt in the basket rather than a large reallocation. For a household near the bottom fifth of the provincial distribution, even a mild tilt leaves room for animal protein and fresh produce in place of cheaper energy-dense staples, which is exactly the shift that the dual-constraint account identified as sensitive to small movements in income and prices. Two controls in column (1) behave as expected and help confirm that the column is reading what it should: per capita income carries −0.021, the familiar pattern of food losing weight in the basket as income rises, and household healthcare expenditure carries −0.018, since medical spending competes for the same budget.

#### 4.3.2 Earnings from going online

Column (2) of Table 6 puts the coefficient of digital access on log labor income at 1.181 with a t-statistic of 1.782, which also reaches the 10% level. The sign points where the economic route says it should, since platform work, online produce sales, and digital microcredit all widen the earnings opportunities available to a household once restricted to the local labor market. Two things nonetheless limit how much weight this column can bear. It rests on 1,128 observations, roughly nine per cent of the working sample, because labor income is recorded only for those who report positive earnings, and those who report earnings are not a random slice of the low-income population. The column is therefore read as directional evidence for H3 and is not set against the other two.

#### 4.3.3 From information to exercise

Column (3) of Table 6 puts the coefficient of digital access on exercise frequency at 0.270 with a t-statistic of 2.021, the clearest of the three. That amounts to roughly one additional session every 4 weeks, a change small enough to be believable and large enough to matter for a group in which most people report no exercise at all. Free health information, dietary guidance, and exercise tutorials circulate online at almost no cost, which lowers the barrier that limited schooling and thin social networks otherwise impose, and regular exercise improves metabolic function at both ends of the weight distribution. This column also carries most of the weight for H2, since the survey holds no direct measure of nutritional literacy and exercise is the behavior most plausibly downstream of better information.

Read together, the three columns of Table 6 line up with H2 through H4 without being equally firm. Exercise frequency stands out most clearly, at the 5% level, while the weight of food in the budget and labor income both point the expected way at the 10% level, and the income result rests on a small group of respondents. The three coefficients sit on three different scales, namely a share, a logarithm, and a weekly count, so no attempt is made to rank the routes by size. What the evidence supports is three consistent signals of differing strength, not a settled account of how much each contributes.

### 4.4 Heterogeneity analysis

An average holds for the sample as a whole and for no one in particular. Given the urban and rural divide, the generational gap in digital skills, and disparities in schooling, there is good reason to expect the link to fall unevenly across the low-income population. Three grouped comparisons follow.

#### 4.4.1 Rural and urban residence

Table 7 splits the sample by place of residence, and the two columns sum to 12,051 rather than the full working sample because 25 observations belong to people who moved between rural and urban residence during the survey period and are left with a single year in one subsample. Among rural residents the coefficient is −0.096 at the 1% level. Among urban residents it is 0.012, and nothing can be concluded from it. Since the rural figure is larger than the pooled one, rural residents carry most of what the overall result reports.

**Table 7:** Heterogeneity by residence.

Variable	(1)	(2)
	NUTRIIMB	NUTRIIMB
	Rural residence	Urban residence
INTERNET	−0.096^***^	0.012
	(−2.801)	(0.134)
EDUYR	−0.009	−0.035
	(−0.766)	(−0.774)
MARRIED	0.167	0.146
	(1.514)	(0.693)
EMPLOYED	−0.073	0.032
	(−1.295)	(0.296)
SRH	−0.011	−0.021
	(−0.747)	(−0.696)
CHRONIC	0.087^**^	−0.114
	(2.177)	(−1.611)
INSURED	0.097	0.021
	(1.143)	(0.156)
RURALHK	−0.150^*^	−0.218^**^
	(−1.646)	(−2.240)
FAMSIZE	0.001	0.031
	(0.066)	(0.830)
LNINCPC	0.017	−0.038
	(0.774)	(−0.699)
LNFOODPC	0.014	−0.026
	(1.002)	(−0.625)
LNASSET	−0.008	0.034
	(−0.477)	(0.988)
CHILDRATIO	−0.082	−0.235
	(−0.530)	(−1.164)
ELDRATIO	−0.045	−0.097
	(−0.367)	(−0.462)
LNMEDEXP	−0.014^**^	−0.013
	(−2.396)	(−1.004)
AGRIOP	0.048	−0.271
	(0.396)	(−1.338)
Observations	8,975	3,076
R-squared	0.839	0.893
Individual FE	YES	YES
Year FE	YES	YES

Cluster-robust *t*-statistics at the individual level in parentheses. ^***^p < 0.01, ^**^p < 0.05, ^*^p < 0.1.

This cuts against a first intuition that digital resources pay off first where other resources are already dense, and it fits a simpler logic instead, namely that connectivity adds most where offline channels are thinnest. Three differences support that reading. Urban low-income residents already live inside a dense offline network of supermarkets, community clinics, wet markets, and in-person health talks, so going online largely duplicates what they have. In rural areas, township health centres and community health education cover far less ground, so the internet becomes a primary low-cost route to information on nutrition, prices, and work. Food procurement differs in the same direction, since urban food retail is dense while rural offline supply is thin and intermediaries raise prices, so buying online widens the rural choice set more. Earnings differ too, since urban low-income residents already have non-agricultural work available and platform income merely supplements it, while rural incomes lean on traditional agriculture and online sales channels matter far more at the margin.

#### 4.4.2 Age

Age divides the low-income population in much the same way that residence does, since it separates those who grew up with the internet from those who came to it late. Table 8 splits the sample at age 50. For adults aged 50 and over the coefficient is −0.076 at the 10% level, and for those under 50 it is −0.065, which falls short of conventional levels. The two figures are close, so what separates them is not a gulf in size, and the reading below is offered in that spirit.

**Table 8:** Heterogeneity by age.

Variable	(1)	(2)
	NUTRIIMB	NUTRIIMB
	Under 50	50 and over
INTERNET	−0.065	−0.076^*^
	(−1.584)	(−1.676)
EDUYR	−0.013	0.003
	(−1.033)	(0.120)
MARRIED	0.193	0.153
	(1.339)	(0.806)
EMPLOYED	−0.112	−0.013
	(−1.348)	(−0.198)
SRH	−0.019	−0.018
	(−0.981)	(−0.906)
CHRONIC	0.218^***^	−0.023
	(3.021)	(−0.584)
INSURED	0.003	0.120
	(0.042)	(0.930)
RURALHK	−0.140^**^	−0.220
	(−1.992)	(−1.639)
FAMSIZE	0.003	0.013
	(0.141)	(0.669)
LNINCPC	−0.027	0.044
	(−1.036)	(1.547)
LNFOODPC	0.000	0.012
	(0.005)	(0.713)
LNASSET	−0.002	0.002
	(−0.100)	(0.080)
CHILDRATIO	0.169	−0.319
	(0.921)	(−1.553)
ELDRATIO	0.055	0.029
	(0.306)	(0.224)
LNMEDEXP	−0.011	−0.018^**^
	(−1.583)	(−2.129)
AGRIOP	−0.104	−0.035
	(−0.562)	(−0.254)
Observations	5,788	6,288
R-squared	0.873	0.848
Individual FE	YES	YES
Year FE	YES	YES

Cluster-robust *t*-statistics at the individual level in parentheses. ^***^p < 0.01, ^**^p < 0.05, ^*^p < 0.1.

Adults aged 50 and over face declining physiological function and a higher prevalence of chronic disease, so both wasting and excess weight are more common among them, and their need for health information is correspondingly greater at a time when community health education more often targets younger residents. Short videos and illustrated health content suit a preference for material that asks little of the reader. Younger adults, by contrast, are already online through most of their daily routine whatever their income, so simply being connected adds less to what they already see, which is consistent with the weaker figure in column (1) of Table 8.

#### 4.4.3 Schooling

If the internet works partly by delivering information, then how easily someone reads should shape what they get out of it. Table 9 splits the sample at 9 years of schooling, so column (1) covers those who went no further than lower secondary education and column (2) covers those who reached senior secondary education or beyond. The two groups are very unequal in size, 10,703 against 1,373, which reflects how little formal schooling low-income adults in this sample completed. Among the lower-schooling group the coefficient is −0.099 at the 1% level. Among the higher-schooling group it is 0.087, and with only 1,373 observations nothing can be read into it either way.

**Table 9:** Heterogeneity by schooling.

Variable	(1)	(2)
	NUTRIIMB	NUTRIIMB
	9 years or less	More than 9 years
INTERNET	−0.099^***^	0.087
	(−3.015)	(0.829)
EDUYR	−0.013	0.075
	(−0.918)	(0.774)
MARRIED	0.115	0.535
	(1.206)	(1.562)
EMPLOYED	−0.032	−0.363^*^
	(−0.627)	(−1.911)
SRH	−0.021	0.064
	(−1.473)	(1.541)
CHRONIC	0.038	0.003
	(1.035)	(0.029)
INSURED	0.085	0.113
	(1.119)	(0.335)
RURALHK	−0.217^**^	0.066
	(−2.518)	(0.797)
FAMSIZE	0.008	−0.024
	(0.584)	(−0.949)
LNINCPC	0.006	0.016
	(0.311)	(0.249)
LNFOODPC	0.007	0.033
	(0.500)	(0.809)
LNASSET	0.003	−0.050
	(0.219)	(−0.784)
CHILDRATIO	−0.092	−0.145
	(−0.701)	(−0.405)
ELDRATIO	−0.016	−0.026
	(−0.152)	(−0.071)
LNMEDEXP	−0.016^***^	0.002
	(−2.768)	(0.193)
AGRIOP	−0.086	0.129
	(−0.785)	(1.020)
Observations	10,703	1,373
R-squared	0.842	0.908
Individual FE	YES	YES
Year FE	YES	YES

Cluster-robust *t*-statistics at the individual level in parentheses. ^***^p < 0.01, ^**^p < 0.05, ^*^p < 0.1.

The pattern points to connectivity working as a partial substitute for schooling. Those who completed lower secondary education at most read printed dietary guidelines and specialist health material with difficulty, so those channels leave the information constraint largely intact. Short videos, illustrated content, and spoken explanation lower the reading requirement, which puts balanced-diet guidance and food pairing within reach without formal study. Those who went further can already search for and interpret specialist material and reach it offline as well, so simply being connected adds less for them.

## 5 Conclusion and implications

Drawing on five waves of the China Family Panel Studies covering 2014 to 2022, this study has examined whether digital access goes together with lower nutritional imbalance among low-income adults and what lies behind that link. Low-income adults who are online carry a lower risk, and the finding survives moving the income line to the 30th and 40th percentiles, narrowing connectivity to mobile devices, and changing the model so that only the ranking of the categories is used. Behind that link, three routes were put to the test. Exercise frequency responds most clearly, at about one extra session every 4 weeks, while the weight of food in the consumption basket rises by roughly 1.4 percentage points and earnings among those who work move in the expected direction, both at the 10% level. The grouped comparisons then show that the benefit falls unevenly rather than across the board, reaching rural residents, adults aged 50 and over, and those with 9 years of schooling or less, while among urban, younger, and better educated low-income adults no clear pattern emerges.

Three implications follow from those findings.

First, digital infrastructure and digital inclusion for low-income groups deserve continued investment. Since the link runs through connectivity itself, expanding coverage in rural and remote areas, lowering broadband and mobile tariffs, and subsidizing devices all act at the point where the link begins. How much this buys on its own should not be overstated, since the basket tilts toward food by only about a percentage and a half, so connectivity alone will not rebuild a diet. Pairing existing nutrition subsidies with e-commerce vouchers restricted to nutrient-dense items such as animal protein and fresh produce would channel that modest tilt toward a better diet rather than a larger grocery bill.

Second, digital tools should be built alongside offline services rather than in place of them. Nothing shows up among urban residents, which is precisely where offline provision is dense, and that pattern indicates that connectivity supplements a service network instead of replacing one. Health education content therefore works best combined with offline check-ups, nutrition programmes, and community support. Since exercise is the behavior that responds most clearly, developers could also be encouraged to offer zero-rated health-promotion applications carrying low-threshold exercise tutorials and dietary guidance, so that changing a habit does not add to the data costs that low-income users already face.

Third, digital support should be aimed squarely at rural residents, adults aged 50 and over, and those with 9 years of schooling or less, the three groups where the link is strongest. For rural areas, nutrition lookup and consultation functions could be built into existing e-commerce service stations at low marginal cost, and produce e-commerce could be joined to agricultural extension so that farmers selling online also receive dietary pairing schemes built around ingredients they can actually obtain locally. For older adults and those with limited schooling, voice interaction and low-literacy interfaces would deliver guidance without asking anyone to read, and digital nutrition education could be added to senior universities and community literacy courses, the offline venues that already reach exactly these groups.

Four limitations qualify what has been reported. First, the outcome rests on body mass index, which gathers long-run dietary and health history into one comparable number but says little about micronutrient deficiency, and the six categories impose an ordering in which the three underweight grades outrank overweight and obesity. That ordering is defensible in a low-income population where being too thin carries the more immediate health penalty, but it is an assumption rather than something the data establish, and a move from obesity to overweight and a move from normal weight to overweight count as steps of the same size. Using only the ranking of the categories relaxes the assumption about spacing but not the ordering itself. Adding dietary intake or biomarker data would allow a finer measure. Second, the route analysis covers the weight of food in the budget, earnings, and exercise, two of which reach only the 10% level, and the earnings result rests on the small group who report positive labor income. The cost of reaching information and the strength of social networks are left unmeasured, and the information route of H2 is read only through behavior that sits downstream of it. Third, the three route variables are observed on groups of different size, so they are not strictly comparable and no attempt is made to apportion the overall link among them. Fourth, the comparisons here follow the same people over time and set aside what is fixed about them and what moves for everyone at once, but they cannot rule out factors that change alongside going online, so the findings are reported throughout as associations.

## Data Availability

The original contributions presented in the study are included in the article/supplementary material, further inquiries can be directed to the corresponding author.
